# Use of Resting Metabolic Rate Ratio as a Relative Energy Deficiency in Sports Indicator in Female Athletes

**DOI:** 10.1016/j.cdnut.2025.106007

**Published:** 2025-03-24

**Authors:** Jessica L Garay, Julia Galindo Sebe, Jenna Strickland, Lindsey Graves, Margaret A Voss

**Affiliations:** 1 Department of Nutrition and Food Studies, Syracuse University, Syracuse, NY, United States; 2 Department of Exercise Science, Syracuse University, Syracuse, NY, United States

**Keywords:** energy availability, sports nutrition, resting metabolic rate ratio, disordered eating, college athlete

## Abstract

**Background:**

Female athletes are at risk of relative energy deficiency in sport (REDs) if energy intake is insufficient relative to demand. REDs is commonly identified via low energy availability (EA), which is determined by measuring dietary intake, exercise energy expenditure, and lean body mass. Due to inconsistent methods to measure each component of EA, the use of resting metabolic rate (RMR) ratio is proposed as an alternate method to identify REDs.

**Objectives:**

The purpose of this study was to identify REDs prevalence among a sample of physically active college-aged females using RMR ratio and correlate this with EA.

**Methods:**

Females (18–24 y) who were members of a NCAA division 1 athletics team or highly physically active (greater than 4 d/wk) participated in the study. Body composition was measured using air displacement plethysmography. RMR was measured using indirect calorimetry. Participants reported dietary intake via a 24-h recall for 1–3 d. EA was calculated using an averaged activity factor of 1.67 to determine exercise energy expenditure. RMR was calculated using standard equations (Harris–Benedict, Owen, and Cunningham).

**Results:**

A total of 77 physically active female college students participated, including 53 NCAA division 1 athletes. Mean EA was 24.5 ± 12.8 kcal/kg fat-free mass/d and 63% of participants met criteria for low EA (<30 kcal/kg fat-free mass/d). Mean RMR ratio was 1.08 ± 0.16, with 19% of the sample having low RMR ratio (below 0.9). Overall, 14% of participants had both low EA and low RMR ratio. There were no differences in EA or RMR ratio between the collegiate athlete and recreational athlete groups.

**Conclusions:**

Physically active female college students, including collegiate athletes, exhibited suboptimal EA. Low RMR ratio appeared to be a more sensitive indicator of REDs risk than low EA.

## Introduction

Among female athletes, particularly those in esthetic and weight-focused sports such as long-distance running, gymnastics, and crew, there is a distinct emphasis on physical appearance and a desire for leanness. This creates a focus on body composition and dietary intake, usually with the goal of losing weight and body fat. In some cases, this may lead to intentional food restriction or other characteristics of disordered eating. Previous research has suggested that collegiate athletes often do not meet their total energy and macronutrient needs [1]. Among female collegiate athletes, only 9% met overall energy needs [2]. Carbohydrates were under consumed, a result also seen in a separate study of female collegiate lacrosse players [2,3]. When dietary intake is chronically insufficient to support exercise needs, an athlete is likely to experience relative energy deficiency in sports (REDs). This condition expands upon the female athlete triad to reflect all impairments an athlete can experience due to energy deficiency, such as a higher risk of injury and poor exercise performance [4].

Athletes who do not consume enough total energy to support their exercise and daily needs are at risk of low energy availability (LEA), typically defined as <30 kcal/kg fat-free mass (FFM)/d [[4], [5], [6]]. LEA is one of the proposed metrics to identify REDs [7]. Measuring energy availability (EA) is challenging due to a lack of a consistent option for direct measurement of exercise energy expenditure (EEE), a key component of the EA calculation. Most previous studies have relied on estimation based on exercise logs or heart rate data. An alternative method of identifying REDs has been proposed using resting metabolic rate (RMR). By comparing measured RMR to calculated RMR, a ratio can be developed. Previous work has suggested that a ratio below 0.9 (or 0.94 in some studies) is indicative of REDs [8]. To our knowledge, few studies have compared prevalence of LEA with that of low RMR ratio among female athletes or looked at associations of both metrics with other components of health.

The primary aim of this study was to evaluate the presence of REDs by determining EA and RMR ratio in a cohort of physically active college-aged females, including collegiate athletes. We hypothesize that prevalence of low EA and low RMR ratio will be similar. A secondary aim of this study was to determine the relationship between body composition, eating disorder risk, and each of these proposed markers of REDs. We hypothesized that athletes with a low body fat and a higher risk of eating disorders will be more likely to have low EA or low RMR ratio.

## Methods

This study was an observational study of a nonrandom sample of physically active female college students. Participants were females, aged 18–24 y, not currently pregnant or breastfeeding, generally healthy (nonsmokers), and self-reported engaging in ≥30 min of physical activity on ≥4 d/wk. Recruitment of participants occurred through e-mails to select student list-servs that included the target population, as well as in-person through presentations to select athletic teams. All participants provided written informed consent prior to beginning data collection. This study was approved by the Syracuse University institutional review board.

Participants had body composition measured using air displacement plethysmography (BOD POD; Cosmed). From this, FFM was obtained for use in the EA calculation (detailed below). RMR was measured using indirect calorimetry (FitMate; Cosmed). This measurement occurred in the morning following an overnight fast. Participants were instructed to perform as little movement as possible in the morning prior to arriving at the laboratory. The measurement lasted 20 min, with the first 5 min discarded. The RMR value was averaged from the final 15 min.

Dietary intake was assessed using an online, self-administered 24-h recall (ASA-24). Participants completed ≥1 recall, but ≤3 recalls (including a maximum of 1 weekend day). If multiple recalls were done, the averaged value was used. Total daily calorie intake was then used for determination of EA as described further. Daily macronutrient intake (grams of carbohydrates, protein, and fat) was determined relative to body size. Participants also completed the Female Athlete Screening Tool (FAST), a 33-item questionnaire validated to identify individuals at risk of disordered eating [9], and the Low Energy Availability in Females questionnaire (LEAF-Q), a comprehensive survey that can be scored to identify individuals at risk of the female athlete triad [10]. The LEAF-Q also asked about total weekly physical activity but did not assess intensity of exercise. Both questionnaires were scored in accordance with previous protocols.

EA was calculated using the following formula: [Dietary intake (kcal) − EEE (kcal)]/FFM (kg)] [11]. EEE was determined by applying the total daily energy expenditure (TDEE) equation: TDEE = RMR + thermic effect of feeding + EEE. First, RMR was multiplied by an activity factor to obtain TDEE. Activity factors of 1.6 and 1.725 were used to reflect moderate to vigorous intensity exercise. The averaged value from these 2 TDEE values was used in the final calculation. Next, 10% of RMR was calculated to represent thermic effect of feeding. This amount was added to the RMR, and the subtotal was then subtracted from TDEE. The resulting number represented the EEE, which was used in the abovementioned EA equation. RMR ratio was calculated as measured RMR divided by predicted RMR. The predicted RMR was calculated using the Harris–Benedict, Cunningham, and Owen female athlete version [8,12,13].

During data analysis, participants were classified according to current status as a collegiate or recreational athlete. Based on calculations, participants were also dichotomized according to EA and RMR ratio. Low EA defined as <30 kcal/kg FFM/d and normal EA defined as >30 kcal/kg FFM/d [4]. Low RMR ratio defined as <0.9 on the Harris–Benedict, Cunningham, or Owen female athlete equations, whereas normal RMR ratio was defined as >0.9 [14].

SPSS software (version 28) was used for all analysis. Bivariate correlation analysis was conducted between all continuous variables. Independent-samples *t* testing was used to compare continuous variables (EA, RMR ratio, body fat percentage, macronutrient intake, FAST score, and LEAF-Q score) between the collegiate athlete and recreational athlete groups. Variables unrelated to each measure were also compared between the low EA and normal EA groups and between the low RMR ratio and normal RMR ratio groups. The χ^2^ testing was conducted between dichotomous categorical variables. Significance was set at *P* < 0.05 for all tests.

## Results

A total of 77 participants completed our study, with 53 division 1 athletes. A profile of the full sample, as well as the collegiate athlete and recreational athlete subgroups, is presented in Table 1. Participants were predominantly non-Hispanic (94%) and White/Caucasian (84%), followed by Black (8%), self-described mixed race (6%), and Asian (3%). Sports represented in the collegiate athlete group included crew (*n* = 12), track/cross-country (*n* = 9), volleyball (*n* = 8), field hockey (*n* = 8), lacrosse (*n* = 4), tennis (*n* = 4), ice hockey (*n* = 3), soccer (*n* = 2), cheer (*n* = 2), and basketball (*n* = 1). Sports represented in the recreational athlete group included weight training (*n* = 19), running (*n* = 2), field hockey (*n* = 1), swimming (*n* = 1), and gymnastics (*n* = 1).

**TABLE 1:** Diet and physical characteristics of study sample.

Measure	Full sample (*N* = 77)	Division 1 athlete (*n* = 53)	Recreational athlete (*n* = 24)
Age (y)	19.6 ± 1.3	19.7 ± 1.3	19.4 ± 1.3
Energy availability (kcal/kg FFM)^1^	24.5 ± 12.8	23.4 ± 13.8	26.9 ± 10.5
Dietary intake (kcal)^1^	2086 ± 639	2123 ± 722	2008 ± 413
Relative energy intake (kcal/kg)^1^	31.8 ± 10.6	31.2 ± 11.4	33.2 ± 8.9
Carbohydrate intake (g/kg)^1^	3.7 ± 1.3	3.6 ± 1.4	3.8 ± 1.3
Protein intake (g/kg)^1^	1.5 ± 0.6	1.4 ± 0.6	1.6 ± 0.7
Resting metabolic rate (FitMate)	1577.6 ± 241.6	1622.5 ± 248.9	1478.5 ± 195.0^2^
Resting metabolic rate ratio (Owen female athlete equation)	1.08 ± 0.16	1.07 ± 0.17	1.09 ± 0.16
Body fat % (Bod Pod)	25.0 ± 5.8	24.3 ± 5.3	26.4 ± 6.6
Female Athlete Screening Tool	68.4 ± 12.9	70.2 ± 11.6	64.4 ± 14.8^2^

Results presented as mean ± SD.

1 Sample size for variable, *n* = 72.

2 Difference between groups: *P* < 0.05.

**FIGURE 1:**
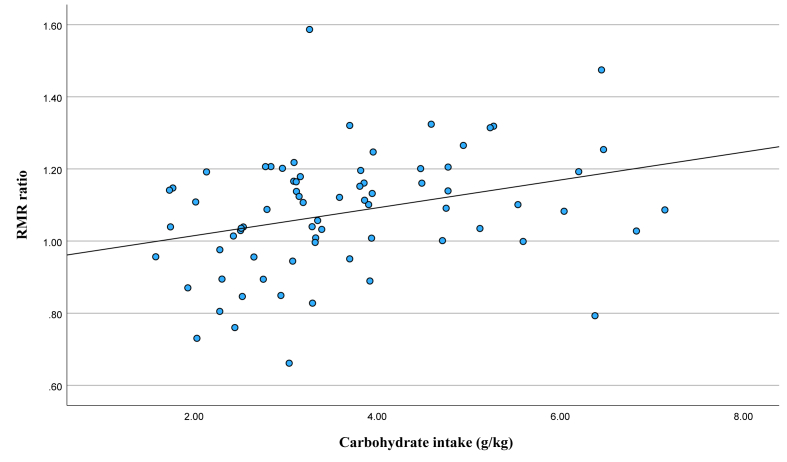
Scatterplot of resting metabolic rate (RMR) ratio and carbohydrate intake; *n* = 72; *R* = 0.315; *P* = 0.007.

RMR ratio (using the Owen female athlete equation) correlated significantly with relative energy intake (*R* = 0.289; *P* = 0.014), protein intake (*R* = 0.319; *P* = 0.006), and carbohydrate intake (*R* = 0.315; *P* =0.007). Figure 1 displays the relationship between RMR ratio and carbohydrate intake. RMR ratio inversely correlated significantly with FAST score (*R* = −0.241; *P* = 0.035), body fat percentage (*R* = −0.277; *P* = 0.015), and FFM (*R* = −0.353; *P* = 0.002). EA was not significantly correlated with any measures, including FAST score, LEAF-Q score, measured RMR, or any of the calculated RMR ratios.

Subclinical eating disorders are identified with FAST scores between 77 and 94, which occurred in 18 participants (23% of sample). There was no difference in presence of subclinical eating disorder risk between the collegiate athlete and recreational athlete groups (χ^2^ = 0.277; *P* = 0.599). Only 1 participant (a recreational athlete) scored above 94, the threshold indicating a possible eating disorder. Among a subsample (*n* = 57) who completed the LEAF-Q, 60% scored an 8 or above, indicating risk of the female athlete triad. There was no difference in LEAF-Q score between the collegiate athlete (*n* =33) and the recreational athlete (*n* = 24) groups (9.4 ± 5.1 and 8.2 ± 4.8, respectively; *P* = 0.196). The number of participants with a LEAF-Q score of ≥8 did not differ between the athlete groups (χ^2^ = 0.518; *P* = 0.472). Interestingly, 5 participants had both a FAST score of >77 and a LEAF-Q score of ≥8, and they were all collegiate athletes. Weekly minutes of exercise differed between groups (1128 ± 396 min/wk for collegiate athletes compared with 690 ± 336 min/wk for recreational athletes; *P* < 0.001). Significantly more recreational athletes reported ever taking a college-level nutrition class than the collegiate athletes (75% compared with 50%; *P* = 0.046).

Low EA was identified in 45 participants (63% of sample). There were no differences in FAST score, LEAF-Q score, body fat percentage, or RMR ratio between the low EA and the normal EA groups. The presence of low EA was not significantly different between the collegiate athlete compared with the recreational athlete group (χ^2^ = 0.607; *P* = 0.436). A total of 15 participants (19% of sample) were identified as having a low RMR ratio. The presence of low RMR ratio was not significantly different between the collegiate athlete compared with the recreational athlete group (χ^2^ = 0.677; *P* = 0.411). Compared with the normal RMR ratio group, the low RMR ratio group had significantly higher body fat percentage (29% ± 7% and 24 ± 5%, respectively; *P* = 0.002), significantly lower protein intake (1.2 ± 0.3 and 1.5 ± 0.7 g/kg body weight/d, respectively; *P* = 0.043), and significantly higher FAST score (74 ± 12 and 67 ± 13, respectively; *P* = 0.034). There was no difference in LEAF-Q score between the RMR ratio groups. In total, 10 participants (14% of sample) likely had REDs due to the presence of both low EA and low RMR ratio.

## Discussion

In our study of physically active college-aged females, including collegiate athletes, we found the prevalence of low EA to occur more frequently than low RMR ratio. In the entire sample, 14% of participants exhibited both low EA and low RMR ratio. There were no differences in occurrence of low EA or low RMR ratio between the collegiate athletes and the recreational athletes. However, across the entire sample, the mean EA was below the threshold for low EA. Participants were highly physically active (960 ± 426 min exercise/wk) compared with general recommendations of 150–300 min of moderate-intensity exercise per week.

Our findings suggest that RMR ratio may be a more sensitive indicator of REDs than low EA, depending on methodology of determining EA and the RMR prediction equations used. After evaluation of several RMR equations, we presented the results using the Owen equation for female athletes, which is in line with prior studies advocating for use of an RMR prediction equation inclusive of FFM rather than overall body weight [8,12,13]. We acknowledge that our method of determining EA may have overestimated EEE based on the activity factors used. However, this highlights the complexity in calculating EA and the variety of approaches used across research, as others have also pointed out [[15], [16], [17], [18]]. Future work should continue to explore options for direct measurement of EEE using wearable devices like the Actiheart. Despite this, we believe that our results suggest an increased likelihood of REDs among physically active young adult females.

Regarding dietary intake, for the entire sample, carbohydrate intake was below typical recommendations for athletes, particularly endurance athletes. Our results agree with prior studies showing insufficient consumption of carbohydrates relative to recommendations for athletes [1,2,19,20]. Given that carbohydrates are typically the largest contributor to total calorie intake, the prevalence of low EA that we observed is likely due to inadequate carbohydrate intake relative to exercise demands.

The FAST questionnaire score, which is used to identify disordered eating patterns, was higher among collegiate athletes than that among recreational athletes. This supports earlier work noting that risk of disordered eating increases with level of competition [21,22]. One previous study found that individuals who strongly identify as an athlete but compete at a lower level have significant risk of disordered eating [23]. The LEAF-Q has previously been shown to positively correlate with FAST scores [24]. In our study, the subgroups who completed the LEAF-Q were insufficiently powered to detect a difference between collegiate and recreational athletes. It should be noted that our sample was drawn from a diversity of sports and activities that place varying levels of emphasis on physical appearance and weight. Other studies have found greater risk of, and prevalence of, disordered eating and eating disorders for female athletes participating in lean or esthetic sports such as gymnastics, dance, and cross-country [21,25,26]. These athletes would benefit from education about energy deficiency and proper fueling. In a separate study conducted by our research team, female collegiate distance runners were found to have inadequate knowledge regarding the female athlete triad or REDs [27]. Those who received education about the female athlete triad recorded better knowledge and confidence about the condition than those without specific education [28].

This study is not without limitations. Participants did not provide specific information about intensity of exercise. Recent participants (*n* = 37) wore an accelerometer device for 4–5 d, which would be used in future analyses to confirm amount of physical activity and intensity level. Regarding dietary intake, ASA-24 does not include sports nutrition-specific food items in the database (ie, gels), making it difficult for athletes to accurately report all foods consumed. Furthermore, we did not track the exact timing of dietary intake relative to time of exercise. Future research may wish to more specifically pinpoint nutrient timing.

Our findings support the need for nutrition education directed at female athletes regarding sufficient carbohydrate and overall energy intake to support exercise and reduce risk of REDs. Previous research has indicated that not only female athletes perceive their total calorie and carbohydrate needs to be lower than recommendations, but their actual intake is also lower than recommendations, even when daily eating patterns include snacks [19,20,29]. Future nutrition education intervention studies targeting female athletes should focus on explaining why carbohydrates are important as a fuel source and provide specific examples of foods containing carbohydrates that athletes could consume before, during, and after exercise. Previous interventions with this population demonstrated positive effects on nutrition knowledge but mixed results on eating behavior and other health-related and performance-related outcomes [[30], [31], [32], [33], [34]]. More research is needed with a variety of collegiate and recreational female athletes. Given the rise of full-time sports dietitian positions within college athletic departments, evaluation of nutrition education directed to individual athletes can now be conducted in a real-world environment.

Finally, the results of this study highlight the importance of REDs awareness for coaches, health professionals, and sport science professionals working with female athletes and collegiate athletes. Previous work by our research team identified that athletic trainers have some knowledge about REDs and the female athlete triad, but this does not always translate to consistent screening for LEA [27]. Education campaigns targeting athletic trainers, strength and conditioning coaches, and other athletic support staff is warranted, especially at organizations lacking a sports dietitian on staff. To our knowledge, such campaigns do not currently exist. One previous study added a nutrition session (delivered by a sports dietitian) to an existing training program for soccer coaches, which led to an increased knowledge and confidence regarding nutrition needs for their athletes [35]. Of course, this also emphasizes the need for sports dietitian positions to exist in all athletic departments, as recommended by a recent consensus statement [36].

In summary, our study found the presence of REDs among physically active college-aged females, including collegiate athletes. The results of this study suggest that low RMR ratio may be a more sensitive method of identifying REDs than low EA, depending on the protocols used to calculate each score. Future research should focus on methods to directly measure EEE and evaluate interventions to educate athletes and health professionals working with athletes about risks associated with REDs and how to appropriately screen for REDs.

## Author contributions

The authors’ responsibilities were as follows – JLG, MAV: designed the research; JLG, JGS, JS, LG: conducted the research; JLG, JS: analyzed data; JLG: wrote the article and had primary responsibility for final content; and all authors: read and approved the final manuscript.

## Data availability

Data described in the manuscript will be made available upon request.

## Funding

This study was supported by the Academy of Nutrition and Dietetics Foundation (Vegetarian Nutrition Dietetic Practice Group) and the Atlantic Coast Conference. Sources of support had no involvement in the study design, data collection, data analysis, or manuscript preparation.

## Conflict of interest

JLG reports financial support by Academy of Nutrition and Dietetics Foundation and Atlantic Coast Conference. The other authors report no conflict of interest.
